# Clinical Significance of Optic Disc Progression by Topographic Change Analysis Maps in Glaucoma: An 8-Year Follow-Up Study

**DOI:** 10.1155/2014/987389

**Published:** 2014-01-21

**Authors:** D. Kourkoutas, Y. M. Buys, J. G. Flanagan, N. Karamaounas, G. Georgopoulos, E. Iliakis, M. M. Moschos, G. E. Trope

**Affiliations:** ^1^Department of Ophthalmology, 401 Hellenic Army General Hospital, 138 Mesogion & Katehaki Avenue, 11525 Athens, Greece; ^2^Department of Ophthalmology and Vision Sciences, University of Toronto, Toronto Western Hospital, 399 Bathurst Street, Toronto, ON, Canada M5T 2S8; ^3^1st Department of Ophthalmology, Medical School, University of Athens, 154 Mesogion Avenue, 11527 Athens, Greece

## Abstract

*Aim*. To investigate the ability of Heidelberg Retina Tomograph (HRT3) Topographic Change Analysis (TCA) map to predict the subsequent development of clinical change, in patients with glaucoma. *Materials*. 61 eyes of 61 patients, which, from a retrospective review were defined as stable on optic nerve head (ONH) stereophotographs and visual field (VF), were enrolled in a prospective study. Eyes were classified as TCA-stable or TCA-progressed based on the TCA map. All patients underwent HRT3, VF, and ONH stereophotography at 9–12 months intervals. Clinical glaucoma progression was determined by masked assessment of ONH stereophotographs and VF Guided Progression Analysis. *Results*. The median (IQR) total HRT follow-up period was 8.1 (7.3, 9.1) years, which included a median retrospective and prospective follow-up time of 3.9 (3.1, 5.0) and 4.0 (3.5, 4.7) years, respectively. In the TCA-stable eyes, VF and/or photographic progression occurred in 5/13 (38.4%) eyes compared to 11/48 (22.9%) of the TCA-progressed eyes. There was no statistically significant association between TCA progression and clinically relevant (photographic and/or VF) progression (hazard ratio, 1.18; *P* = 0.762). The observed median time to clinical progression from enrollment was significantly shorter in the TCA-progressed group compared to the TCA-stable group (*P* = 0.04). *Conclusion*. Our results indicate that the commercially available TCA progression criteria do not adequately predict subsequent photographic and/or VF progression.

## 1. Introduction

Accurate assessment of structural and functional change in glaucoma is important for diagnosis and progression detection. In current clinical practice, functional progression is monitored by standard automated perimetry (SAP). Structural progression is subjectively determined by clinical evaluation of optic nerve head (ONH) stereophotographs [1–4]. Results from the Collaborative Initial Glaucoma Treatment Study (CIGTS) and Ocular Hypertension Treatment Study (OHTS) support the view that optic disc change may be a more sensitive indicator of glaucomatous progression than VF change in patients with glaucoma or ocular hypertension (OHT) [5, 6]. However, in many cases VF defects were the first sign of glaucomatous development in OHT eyes [5].

Objective technologies such as the Heidelberg Retina Tomograph (HRT, Heidelberg Engineering, GmbH, Dossenheim, Germany) have been developed as adjuncts to subjective ONH evaluation. The HRT is a confocal scanning laser tomography device that creates reproducible and repeatable three-dimensional topographic images of the ONH and the peripapillary retina [7].

The HRT software includes a commercially available software package to help evaluate change over time called the Topographic Change Analysis (TCA) map [8, 9].

The TCA map is an event-based technique for detecting topographic surface height changes across the entire ONH and peripapillary surface at a superpixel level between baseline and follow-up images. Progression is identified when the change exceeds measurement variability and is confirmed in, at least, 3 consecutive tests (error probability <5%). In the currently available software (HRT3), progression is defined as a cluster of 20 or more significantly depressed superpixels [9, 10].

There is increasing evidence in the literature that TCA can detect progressive optic disc changes [9, 11–14]. Additionally, eyes with previous optic disc change by TCA map were more likely to have subsequent VF progression [15]. Nevertheless, as our previous study and other studies have demonstrated, there is a significant subset of patients who show TCA changes without ONH stereophotograph [11–13] nor VF progression [13].

The aim of this study was to determine, in a prospective fashion, how well TCA maps are able to predict the subsequent development of ONH and/or VF changes.

## 2. Methods

### 2.1. Patients

This was a prospective cohort study that enrolled clinically stable eyes with glaucoma. Subject recruitment took place in the Glaucoma Unit of Toronto Western Hospital, University Health Network, Toronto, Canada. Data analysis and interpretation took place in the Glaucoma Units of Toronto Western Hospital, and University of Athens, Athens, Greece. In accordance with the Declaration of Helsinki, patients gave informed consent to participate in the study and the protocol was approved by the University Health Network Research Ethics Board.

To identify clinically stable eyes a retrospective chart review of all patients that had undergone ONH scanning with HRT between 1997 and 2006 was performed. Inclusion criteria were the following.

1. Diagnosis of glaucoma: subjects were defined as having glaucoma according to VF defects regardless of the clinical appearance of the optic disc. Glaucomatous VF defect was defined [16] by a Glaucoma Hemifield Test (GHT) outside normal limits on at least two VFs, or a cluster of three or more nonedge points in a location typical for glaucoma, all of which are depressed on the pattern deviation plot at a *P* < 5% level and one of which is depressed at a *P* < 1% level on two consecutive VFs, or a pattern standard deviation (PSD) with *P* < 5% level on two consecutive VFs.
2. Best corrected visual acuity >20/60.
3. >2 years followup.
4. ≥4 good-quality HRT examinations (SD ≤ 50);
5. Reliable baseline VF (≤33% false positives, false negatives, and fixation losses) performed within 8 months of their first and most recent HRT examination.
6. ONH stereophotographs within 8 months of their first and most recent HRT examination.

Exclusion criteria were (1) systemic disease or systemic medication known to affect the visual field; (2) refractive error exceeding 5 diopters (equivalent sphere) of myopia or hyperopia, or 3 diopters of astigmatism; and (3) concomitant eye disease. If both eyes were eligible, one eye was randomly chosen.

**Figure 1:**
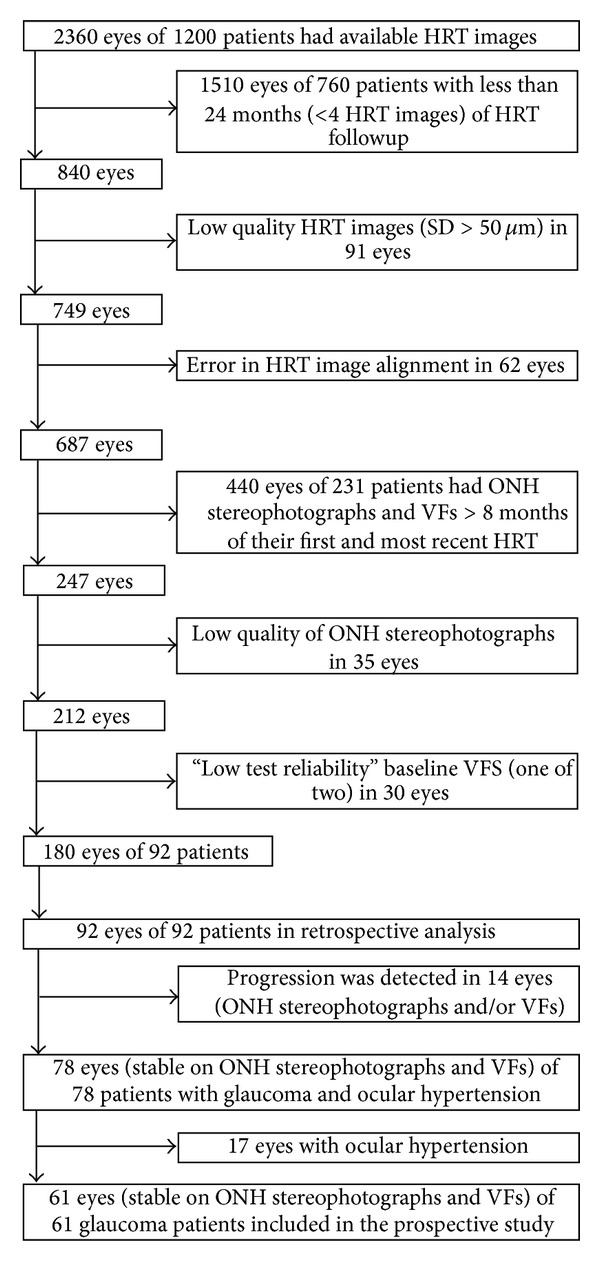
Flow chart showing study exclusion process.

From a total of 1200 patients, 92 eyes of 92 patients met the above criteria. ONH stereophotographs and VF tests of those 92 patients were retrospectively evaluated to identify if they had VF and/or photographic change (Figure 1).

After this initial retrospective evaluation, 61 eyes of 61 glaucoma patients were identified as clinically stable (stable on both ONH stereophotographs and VFs) and were enrolled in a prospective study. At enrollment in the prospective study, 27/61 clinically stable eyes were classified as *TCA-*stable and 34/61 clinically stable eyes were classified as* TCA-*progressed according to HRT3 progression criteria. During the prospective period all patients underwent testing with HRT3, SAP, and ONH stereophotographs at 9–12 months intervals.

Treatment changes were made in order to maintain target IOP. As there is no published evidence to show TCA's benefit regarding initiation or change to treatment regimes, no treatment changes were initiated based on TCA indications for progression alone [11, 17].

### 2.2. Glaucoma Progression

The total followup consisted of an initial (retrospective or preenrollment) and a subsequent (prospective) followup with the objective of determining how TCA change during the initial follow-up predicted clinical progression during the subsequent followup. For all three testing methods used in this study, progression was determined by comparing the most recent examination to the initial baseline using the progression criteria described below. In the current study, clinical progression was defined as a worsening on VFs and/or ONH stereophotographs by the following criteria.

#### 2.2.1. HRT ONH Imaging

While HRTII software was used for image acquisition in the preenrollment period, HRT3 was used for image acquisition in the prospective period. HRT image analysis, throughout the study period (retrospective and prospective period), was performed by HRT3 software. According to currently available TCA progression criteria, progression was defined based on the largest cluster of repeatable depressed superpixels (>20 superpixels) [10] with statistically significant change within the optic disc-cluster totally or partially inside the ONH margin. Progression was not considered when the clusters of superpixels were observed on ONH vessels [18, 19]. Additionally, HRT images were checked for good alignment. Poor quality (SD > 50 *μ*m) and misaligned images were excluded and the whole series reprocessed. Throughout the study, eyes were classified as *TCA-*progressed or *TCA-*stable based on the HRT3 TCA results.

#### 2.2.2. Stereoscopic Optic Disc Photography

All stereophotographs were sequentially obtained with the same model fundus camera (Topcon retinal camera TRC.50IX) by experienced technicians. Progressive change in stereophotographs (focal or concentric rim thinning, increased vertical cup-to-disc ratio, new or enlarged RNFL defect, or the presence of a new disc hemorrhage) was defined as a clinical change in the ONH.

Evidence of progression was based on masked comparison between the baseline and most recent stereophotograph, independently by two experienced glaucoma specialists (Trope and Buys) [11, 20]. Observers were masked to patient identity, diagnosis, and the other observer's results. The temporal order of each photo pair was unmasked. Progression was defined as a dichotomous variable (progression versus no progression). Additionally, the observers were asked to indicate the location where change had occurred. If the observers disagreed, a consensus evaluation was undertaken.

#### 2.2.3. Visual Field Testing

VFs were assessed for progression by using criteria from the Early Manifest Glaucoma Trial [21]. The Humphrey Guided Progression Analysis (GPA) was used [22].

Progression on VFs was defined as “likely progression” based on SAP GPA, requiring significant change in ≥3 points in 3 consecutive follow-up VFs.

#### 2.2.4. Statistical Analysis

Quantitative characteristics of the study population were summarized through median and interquartile range (IQR), whereas absolute and relative frequencies were used to summarize categorical variables. Descriptive characteristics were presented according to the TCA status at enrollment date in the prospective study (Progressed or Stable). Comparisons between these two groups were performed using Mann-Whitney *U* tests and Fisher's exact tests for quantitative and qualitative characteristics, respectively.

Differences in time-to-clinical (stereophotograph and/or VF) progression according to TCA status were evaluated through survival analysis techniques. More specifically, log-rank tests and Cox proportional hazards models were used to evaluate the prognostic value of TCA status along with other factors.

Probability of clinical progression was estimated and plotted using the Kaplan-Meier method. The focus of this study was to see if TCA progression or TCA stability had any influence on the subsequent development of clinically detectable glaucomatous change. Therefore, enrollment date in the prospective study (time-point when TCA status became known) was used as the time origin for the survival analyses. The TCA status was used as a time-updated variable (time-varying covariate).

Interobserver reliability with respect to stereophotograph evaluation was calculated by Cohen's Kappa statistic; [23] The Bias index (BI), the Prevalence index (PI), the Bias Adjusted Kappa (BAK), and the Prevalence and Bias Adjusted Kappa (PABAK) were also calculated [23–25].

A power analysis was performed to determine the smallest hazard ratio for clinical progression that could be detected with the current study size. For the primary outcome of VF and/or ONH stereophotograph progression, given the number of TCA stable and TCA-progressed eyes at enrollment and assuming an annual progression rate of 0.08 in the TCA stable group (similar to EMGT) [26], the smallest hazard ratio that could be detected with at least 80% power (at *α* = 0.05 by 2-tailed test) was 3.3.

## 3. Results

Sixty-one clinically stable eyes of 61 patients with glaucoma were included. Each study eye was classified as *TCA-*stable or *TCA-*progressed. Demographic and clinical characteristics of study patients/eyes are shown in Tables 1 and 2. At baseline, 53/61 eyes had early VF defects (MD >−6.0 dB), 6/61 eyes had moderate VF defects (−6.0 dB > MD >−12.0 dB), and 2/61 eyes had advanced VF defects (MD <−12.0 dB) (Table 2).

Agreement between the two observers in the evaluation of stereophotographs was moderate (proportion of observed agreement *P*_o_ = 0.90 (95% confidence interval, CI): 0.86–0.94), with Cohen's kappa coefficient (*k*) = 0.43 (95% CI: 0.23–0.63). The BI was −0.014, the PI was 0.81, the BAK was 0.43, and the PABAK was 0.81.

The median (IQR) absolute time difference between the baseline HRT examination and baseline disc photograph was 0.27 (0, 0.87) months, and the baseline HRT examination and baseline VF was 0.23 (0, 1.37) months. The median (IQR) total HRT followup was 8.1 (7.0, 9.1) years, which includes a median preenrollment (or retrospective) and prospective follow-up time of 3.9 (3.1, 5.0) and 4.0 (3.5, 4.7) years respectively (Table 2). There were no statistically significant differences in enrollment characteristics between TCA stable and TCA-progressed eyes (Table 2).

Quality of HRT images was very good throughout the total follow-up period (median (IQR) HRT topography SD was 17 (13, 22)). Of the approximately 840 HRT images, only 7 had a SD between 40 and 50 *μ*m. The median (IQR) HRT topography SD in the TCA-progressed group was 17 (13, 22) and in the TCA-stable group was 16 (12, 19). A Kruskal-Wallis analysis was performed that indicated a nonsignificant difference between the two groups (H = 4.318, 1 d.f., *P* = 0.038).

TCA progression was documented in 48/61 (78.7%) eyes during the study but clinical progression (ONH stereophotograph and/or VF change) was detected in only 16/61 (26.2%) eyes.

**Figure 2:**
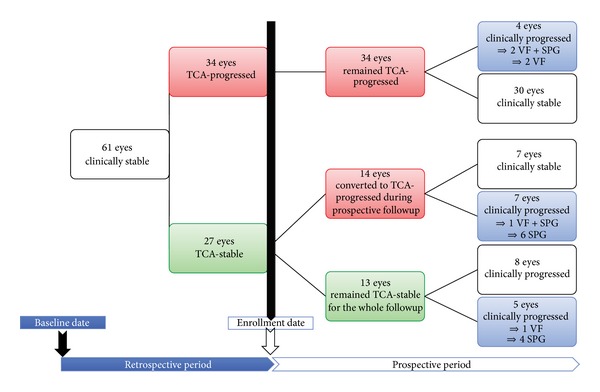
Study flow chart. VF: visual field; SPG: stereophotograph; TCA: Topographic change analysis.

Figure 1 shows the study selection and exclusion process and Figure 2 details the prospective period in which clinical progression (photographic ONH and/or VF change) occurred in 5/13 (38.4%) TCA-stable eyes and in 11/48 (22.9%) TCA-progressed eyes.

**Figure 3:**
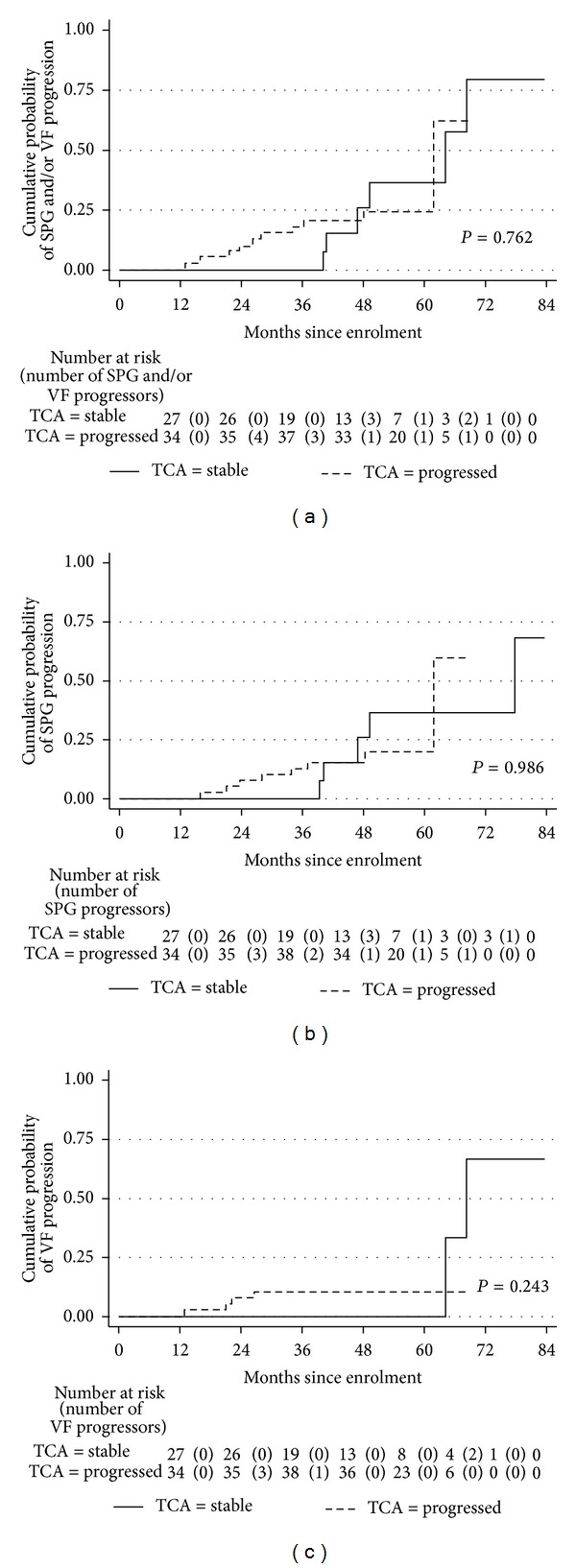
Kaplan-Meier curves for stereophotograph (SPG) and/or visual field (VF) progression (a), SPG only progression (b), and VF only progression (c), from recruitment, by topography change analysis (TCA) status. *P* values are from log-rank test.

The Kaplan-Meier cumulative probability of stereophotograph and/or VF progression in eyes with previous TCA progression was 18%, 20.5%, and 24.4% by 3, 4, and 5 years after enrollment, respectively, compared with 0.0%, 26%, and 36.5% in TCA-stable eyes (Figure 3, Table 5).

Table 3 reports the hazard ratios (HRs) with 95% confidence intervals (CIs) from univariate Cox models for each potential predictive factor for glaucoma progression. TCA progression was not associated with a statistically significant increased risk of photographic and/or VF progression compared with TCA stability (HR, 1.18; *P* = 0.76). For any fixed point in time, TCA-progressed eyes were at nearly four times the risk of VF progression as TCA-stable eyes; however, this was not statistically significant (HR 3.70; *P* = 0.27).

The median survival time could not be computed because less than half the subjects reached the event of interest (stereophotograph and/or VF progression). Instead the observed median times to stereophotograph and/or VF progression were calculated, for those who showed stereophotograph and/or VF progression. The observed median time to VF and/or stereophotograph progression from enrollment was statistically significant shorter in the TCA-progressed group compared to the TCA-stable group (Table 4).

In 9 eyes of our cohort, HRTI scans were used as baseline images. Survival analyses were also performed, after excluding the 9 eyes with HRTI baseline, which gave similar results. More in detail, the Kaplan-Meier cumulative probability of stereophotograph and/or VF progression in eyes with previous TCA progression was 20.6%, 23.4%, and 28.2% by 3, 4, and 5 years after enrollment, respectively, compared with 0.0%, 28.6%, and 40.5% in TCA-stable eyes (log rank *P* = 0.83). TCA progression was not associated with a statistically significant increased risk of photographic and/or VF progression compared with TCA stability (HR, 1.14; *P* = 0.82).

## 4. Discussion

TCA-map has shown good ability to detect progressive ONH changes [9, 11–14]. However, the clinical usefulness of commercially available TCA progression criteria [10] is significantly limited since a large subset of patients with ONH changes on TCA has no clinically detectable change (22.2% to 55.4% of TCA progressing eyes) [9, 11].

In the present study, we investigated whether eyes with TCA progression at enrollment, but no VF and ONH stereophotograph change, were associated with increased risk of subsequently developing clinically detectable glaucoma progression based on VF GPA and expert assessment of ONH stereophotographs. For this reason, we included eyes that had neither photographic ONH nor functional progression for a median retrospective follow-up period of 3.9 years (Table 2). In addition, we should note the fact that we mostly enrolled clinically stable eyes with early glaucomatous VF damage (Table 2)—as a result of the patient selection process and the availability of eligible patient—and, therefore, our results may not apply to glaucoma patients in general.

Although SAP and ONH stereophotographs may be imperfect reference standards, they are widely used in clinical practice and have been validated by major clinical trials [27–29]. To the best of our knowledge, our study is the first publication on ONH stereophotograph and VF progression after longitudinally observed TCA change in patients with stable glaucoma.

Our results indicate that current TCA progression criteria [10] do not successfully predict subsequent photographic and/or VF progression as defined in this study. The predictive ability of TCA maps did not significantly improve when different criteria were used to define progression (disc stereophotographs, GPA, disc stereophotographs, and/or GPA) (Figure 3, Table 5). The survival curves reveal that, after 3 years of prospective followup, the TCA-progressed group had a nonstatistically significant higher probability of photographic and/or VF progression (Figure 3, Table 5).

**Figure 4:**
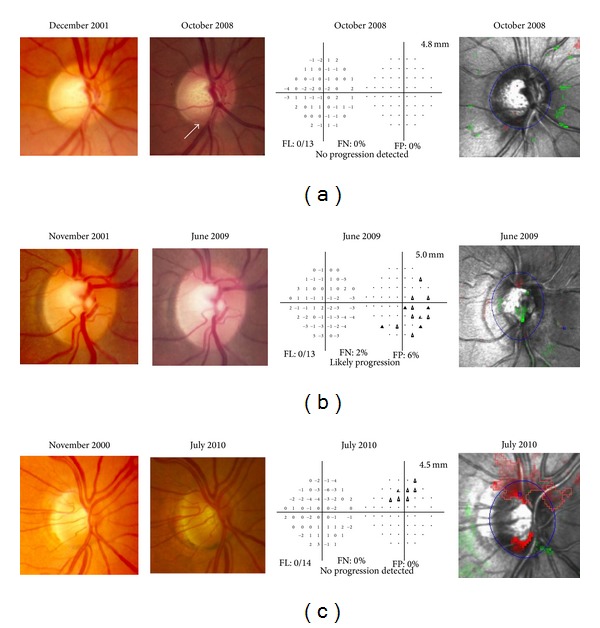
Case examples of disagreement for detecting glaucoma progression among Topographic Change Analysis (TCA) map, optic nerve head (ONH) stereophotographs (SPGs), and Visual Fields (VFs). (a) Low tension glaucoma (LTG), OD, female. This eye was TCA-stable at recruitment (2001). During the prospective followup it remained stable on both TCA and VF. Progression was found only on SPG (increased cupping and inferior rim thinning). (b) Primary open angle glaucoma (POAG), OD, female. This eye was TCA-stable at recruitment (2001). During the prospective followup it remained stable on both TCA and SPG. Progression was found only on VF. (c) POAG, OD, female. This eye was TCA-progressed at recruitment (2000). During the prospective followup it remained stable on both VF and SPG. TCA showed progression (superior and inferior rim thinning) throughout the study period.

Our previous work [11] tried to identify discrepancies between TCA-progression and progression detected by expert-assessed sequential disc stereophotographs. This 2.6 year retrospective study on 54 patients reported agreement in only 65% of cases. A smaller proportion progressed on stereophotograph assessment (6%, or 3 eyes) compared to TCA (30%, or 16 eyes). Other studies [9, 12] comparing longitudinal TCA and stereophotographs have also reported that agreement between these 2 structural assessments is moderate, with concordances of 81% and 44% to 71% (depending on progression criteria). This could be attributed to the fact that different methods possibly provide complimentary progression information as illustrated in our cases of disagreement (Figure 4).

Table 3 details univariate Cox's models to investigate the relationship between clinically relevant glaucoma progression and patient characteristics. TCA progression was associated with an insignificant trendto increased risk for clinically relevant glaucoma progression. All other evaluated risk factors showed no significant prognostic effect. This result could be due to the relatively small number of clinically progressing eyes (16/61, 26.2%) in our cohort.

Data in Table 4 indicates that TCA change may be associated with earlier clinical glaucoma progression. More in detail, the observed median time to VF and/or ONH stereophotograph progression from enrollment was significantly shorter in the TCA-progressed group compared to the TCA-stable group (*P* = 0.04). Given the small numbers, the quality of available evidence is low and not suitable for drawing conclusions. Chauhan et al. [15] recently showed, by using a conservative TCA criterion, that mean time to VF progression was statistically significantly shorter in patients with previous TCA disc changes. Nevertheless, the predictive ability of TCA progression was found to be rather weak with more than 70% of the TCA progressing eyes not developing VF progression when followed for more than 6 years after TCA progression was detected [15].

**Figure 5:**
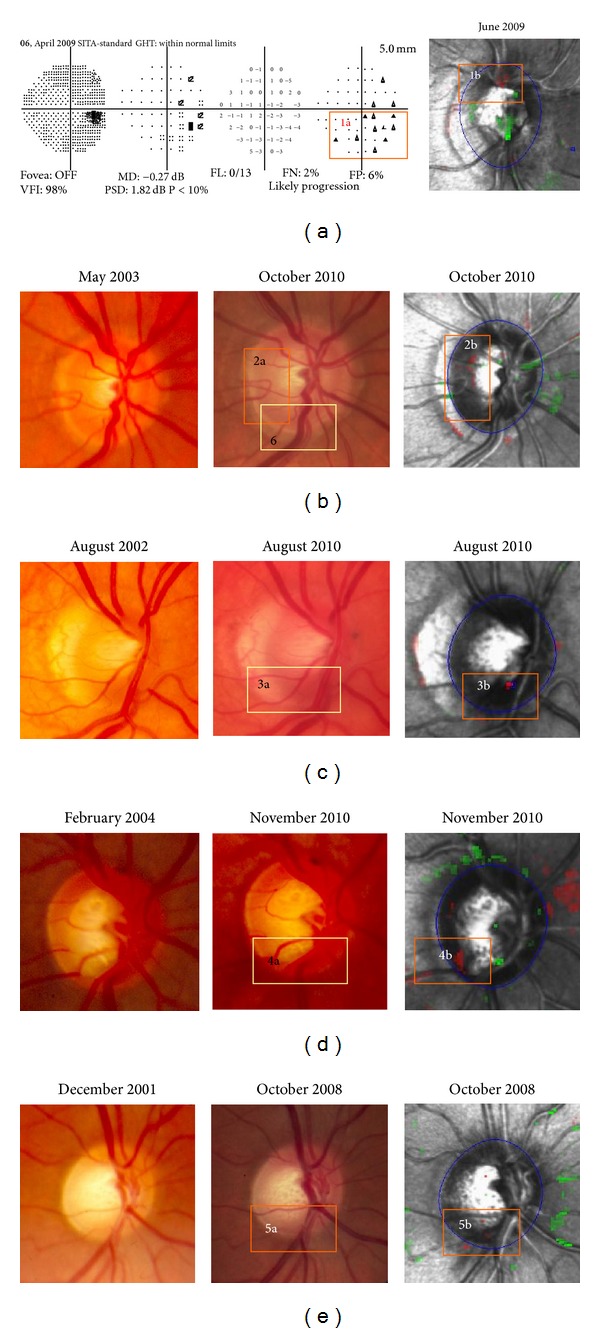
Five out of 13 TCA-stable cases showing clinical progression. In all cases the HRT highlighted the corresponding optic nerve head (ONH) area but with fewer than 20 superpixels. (a) Progression was found on inferior visual field (VF) hemifield (1a). The HRT highlighted the corresponding ONH area—over the vessels (1b). (b) Progression was found on stereophotograph (SPG) (temporal (2a) and inferior (6) rim thinning). TCA highlighted the temporal ONH area only (2b). (c), (d) and (e) Clinical progression in 3a, 4a, and 5a disc areas was highlighted by TCA (3b, 4b, and 5b, resp.).

Of concern was the fact that a significant number of TCA stable patients (5/13, 38.4%) showed photographic ONH or VF progression. In each case, TCA identified the corresponding ONH area as being abnormal but with fewer than 20 red superpixels (Figure 5). This suggests that in these cases the TCA criteria were too conservative and may need adjustment based on other factors such as disease severity or location to detect progression.

Several different methods have been proposed to improve the performance of the TCA technique to monitor progression, which are yet to be incorporated into the operational software. These include using TCA cutoffs, statistical image mapping (SIM) technique, and proper orthogonal decomposition [13, 30, 31]. O'Leary et al. [32] compared change in HRT image series identified by 3 automated statistical analytical methods (TCA, SIM, and ordinary least squares linear regression of rim area (RALR)) with optic disc stereophotographs assessments by glaucoma specialists. This study showed poor agreement between glaucoma expert-assessed stereophotographs and the 3 HRT statistical analyses. At a fixed specificity of 90% for all 3 methods, sensitivities were 25% for TCA, 27% for SIM, and 40% for RALR.

Bowd et al. [13] introduced new TCA parameters and suggested parameter cutoffs for detecting progression in eyes with suspected or known primary open angle glaucoma. When the best performing cutoffs were applied to longitudinal topographic series obtained from patient eyes observed for four or more years and showing no evidence of progression based on SAP or stereophotographic assessment, specificities were poor to moderate (from 0.464 for *CAREA*disc (area of a cluster, in mm^2^, within the optic disc margin), with the 0.90 specificity cutoff of 0.036 mm^2^, to 0.647, with the 0.99 specificity cutoff of 0.074 mm^2^).

There are certain limitations that need to be acknowledged and addressed regarding the present study. One limitation of this and similar studies is the lack of a sufficiently independent valid reference standard for progression. We decided to employ structural and functional endpoints despite the fact that the comparison of an imaging technology is likely more relevant to photographic optic disc progression rather than perimetry. The EMGT criterion for VF progression was recently shown to have high specificity [28]. Nevertheless, it may be the case that true progression is missed using subjective stereophotograph assessment. Also, it is likely that VF progression does not occur in temporal conjunction with structural change, regardless of progression criteria.

Our observers' agreement reviewing ONH stereophotographs has been evaluated in previous studies [11, 20] and is consistent with other studies [33–35]. Despite an interobserver agreement of 90% (proportion of observed agreement *P*_o_ = 0.90 (95%, CI): 0.86–0.94), the kappa value (kappa = 0.43) suggests moderate agreement. However, the PABAK (0.81) revealed that kappa (0.43) was influenced by the low prevalence of disc change as judged by either observer and therefore it may be erroneous to conclude that our observers' ratings were unreliable [36].

Another limitation of this study is the fact that TCA progression criteria included statistically significant clusters of red superpixels located either totally or partially inside the ONH margin. Clusters lying mostly outside the ONH margin could probably not be detected clinically by looking at optic disc stereophotos. Therefore, such a criterion could induce false positive TCA-progressed patients. Nevertheless, the commercially available HRT3 software, we used in the current study, does not have the ability to delineate a significant cluster of superpixels within the ONH margin if it belongs to a larger cluster that is extended partially outside the optic disc as well.

A possible weakness of this study is that the HRT software was updated during the 8-year followup. Image acquisition was performed by using HRTII in the preenrollment period and HRT3 in the prospective period. In 9 eyes, HRTI scans were used as baseline images. Image analysis however was carried out in both pre-enrollment and prospective periods by using only HRT3. The HRT3 has the advantage of compatibility with the earlier versions allowing glaucoma progression to be detected over a much longer time period [37]. Recent evidence shows that in some eyes, TCA progression may be overestimated when HRTI scans are used as baseline images with HRTII scans as follow-up images [38]. In our cohort, results from the survival analyses were similar after excluding the 9 eyes with HRTI baseline.

Possible limitation also includes the fact that the current study was powered to detect a large HR (HR ≥ 3.3) for development of clinical progression. Given the fact we did not find that the expected association raises the possibility that this could have been due to a small sample size and thus our evaluations were possibly underpowered. This is also suggested by the fact that no other risk factor was associated with clinical progression in the Cox proportional hazards analysis. Nevertheless, the HR magnitude that could be detected by our study size was generally lower compared to hazard ratios for the association between other HRT parameters (moorfields regression analysis (MRA) and Glaucoma Probability Score (GPS)) and the development of glaucoma progression in the OHTS. Weinreb et al. [39] reported hazard ratios ranging from 2.92 to 3.59 for the GPS global and regional parameters outside normal limits compared with within normal limits, and from 3.34 to 14.25 for the MRA global and regional parameters outside normal limits compared with not outside normal limits.

In conclusion, the identification of clusterwise significant change needs clinical interpretation and possibly redefinition of current progression criteria in order to take advantage of the many practical advantages of the HRT. Our results suggest that TCA progression on its own was associated with only a statistically insignificant trendto increased risk for clinically relevant glaucoma progression. It is possible that TCA ONH change may be associated with earlier clinical glaucoma progression but longer followup is required to prove this. In addition, clinical change was found in nearly 40% of eyes deemed stable by TCA. Although TCA maps are widely used there are currently only few suggestions as to what constitutes clinically relevant change. Therefore, clinicians should not rely on a single method to determine disease progression.

## Figures and Tables

**Table 1:** Descriptive characteristics of study eyes/patients by HRT TCA status at enrollment.

	TCA status at enrollment			
	TCA-stable *n* (%)	TCA-progressed *n* (%)	Overall *n* (%)	*P* value
Total	27 (100.0)	34 (100.0)	61 (100.0)	
Eye				0.43
OD	20 (74.1)	22 (64.7)	42 (68.9)	
OS	7 (25.9)	12 (35.3)	19 (31.1)	
Sex				0.89
Male	13 (48.1)	17 (50.0)	30 (49.2)	
Female	14 (51.9)	17 (50.0)	31 (50.8)	
Glaucoma type				0.96
POAG	14 (51.9)	18 (42.9)	32 (52.5)	
LTG	8 (29.6)	9 (26.5)	17 (27.9)	
CACG	2 (7.4)	2 (5.9)	4 (6.6)	
PG	2 (7.4)	3 (8.8)	5 (8.2)	
MMG	1 (3.7)	1 (2.9)	2 (3.3)	
Axenfeld syndrome	0 (0.0)	1 (2.9)	1 (1.6)	
HRT MRA (at baseline)				0.27
Within normal limits	7 (25.9)	14 (41.2)	21 (34.4)	
Borderline	6 (22.2)	6 (17.6)	12 (19.7)	
Outside normal limits	14 (51.9)	14 (41.2)	28 (45.9)	

POAG: primary open angle glaucoma; LTG: low tension glaucoma; CACG: chronic angle closure glaucoma; PG: pigmentary glaucoma; MMG: mixed mechanism glaucoma; MRA: moorfields regression analysis.

**Table 2:** Comparison of baseline and enrollment descriptive characteristics, in TCA-progressed (based on HRT TCA status at enrollment) and TCA-stable eyes/patients.

	TCA status at enrollment			
	TCA-stable	TCA-progressed	Overall	*P* value
	Median (IQR)	Median (IQR)	Median (IQR)	
Age at baseline (yrs)	55.3 (51.0, 61.6)	57.1 (53.3, 60.8)	56.5 (51.4, 60.8)	0.39
Age at enrollment (yrs)	59.2 (54.2, 65.5)	60.7 (58.3, 65.5)	60.3 (56.4, 65.5)	0.29
Preenrollment followup (yrs)	3.5 (2.8, 4.5)	4.4 (3.2, 5.3)	3.9 (3.1, 5.0)	0.09
VF exams, preenrollment period (*n*)	5 (4, 6), range: 3–8	5 (4, 6), range: 3–11	5 (4, 6), range: 3–11	0.16
VF exams, prospective period (*n*)	7 (6, 8), range: 3–13	7 (5.25, 8), range: 3–9	7 (6, 8), range: 3–13	0.36
HRT exams, preenrollment period (*n*)	6 (5, 6), range: 4–10	6 (5, 7), range: 4–10	6 (5, 7), range: 4–10	0.11
HRT exams, prospective period (*n*)	7 (5, 8), range: 2–11	7 (6, 8), range: 2–10	7 (6, 8), range: 2–11	0.14
CCT (*μ*m)	550.0 (525.0, 577.0)	548.0 (531.0, 565.0)	548.0 (530.0, 570.0)	0.96
MD at baseline (dB)	−1.1 (−2.0, 0.1)	−1.6 (−3.5, 0.0)	−1.2 (−3.1, 0.0)	0.37
PSD at baseline (dB)	1.6 (1.3, 2.5)	1.9 (1.4, 2.8)	1.8 (1.4, 2.6)	0.22
VFI at baseline (%)	99.0 (98.0, 100.0)	98.0 (95.0, 99.0)	99.0 (96.0, 100.0)	0.17
GHT at baseline	1.0 (1.0, 3.0)	2.0 (1.0, 3.0)	1.0 (1.0, 3.0)	0.32
MRA at baseline	2.0 (1.0, 3.0)	2.0 (1.0, 3.0)	2.0 (1.0, 3.0)	0.27
MD at enrollment (dB)	−0.78 (−2.64, 0.15)	−0.99 (−2.7, 0.08)	−0.80 (−2.79, 0.03)	0.48
PSD at enrollment (dB)	1.53 (1.39, 2.08)	1.53 (1.39, 2.08)	1.77 (1.4, 2.4)	0.06
VFI at enrollment (%)	99 (98.5, 100)	98 (96, 99)	99 (97, 99)	0.09
Mean HRT topography SD at baseline (µm)	17 (13.5, 22.5)	16 (11.0, 21.0)	16 (12, 21)	0.27

IQR: interquartile ranges; CCT: central corneal thickness; VF: visual field; MD: mean deviation; PSD: pattern standard deviation; VFI: visual field index; SD: standard deviation; Stdev: standard deviation; GHT: glaucoma hemifield test (1.0: within normal limits, 2.0: borderline, and 3.0: outside normal limits); MRA: moorfields regression analysis (1.0: within normal limits, 2.0: borderline, and 3.0: outside normal limits).

**Table 3:** Univariable Cox models for the hazard of glaucoma progression by TCA status and other potential prognostic factors.

Covariate	Stereophotograph and/or Visual Field			Stereophotographs			Visual Fields		
	HR	95% CI	*P*	HR	95% CI	*P*	HR	95% CI	*P*
TCA progression									
Stable	1.00			1.00			1.00		
Progressed	1.18	(0.39, 3.63)	0.76	0.99	(0.29, 3.36)	0.99	3.70	(0.37, 37.14)	0.27
Sex									
Male	1.00			1.00			1.00		
Female	1.52	(0.54, 4.33)	0.43	1.53	(0.48, 4.84)	0.47	3.56	(0.39, 32.63)	0.26
MRA at baseline									
Within normal limits	1.00			1.00			1.00		
Borderline	1.56	(0.36, 6.72)	0.55	2.35	(0.47, 11.73)	0.30	1.02	(0.10, 10.81)	0.99
Outside normal limits	1.05	(0.34, 3.24)	0.94	1.31	(0.33, 5.32)	0.70	0.45	(0.07, 2.76)	0.39
CCT (per 50 *μ*m thicker)	0.85	(0.46, 1.58)	0.61	0.90	(0.47, 1.74)	0.76	0.48	(0.16, 1.44)	0.19
MD at baseline (per 1 dB larger)	1.03	(0.88, 1.21)	0.69	1.08	(0.89, 1.32)	0.43	0.93	(0.75, 1.16)	0.53
PSD at baseline (per 1 dB larger)	1.03	(0.91, 1.17)	0.63	0.99	(0.85, 1.16)	0.92	1.13	(0.94, 1.37)	0.19
VFI at baseline (per 10 units)	0.99	(0.57, 1.73)	0.97	1.11	(0.56, 2.19)	0.77	0.77	(0.33, 1.78)	0.54
Age at baseline (per decade)	1.11	(0.62, 1.97)	0.73	1.13	(0.59, 2.16)	0.70	1.91	(0.54, 6.76)	0.31
Age at enrollment (per decade)	1.12	(0.64, 1.99)	0.68	1.15	(0.61, 2.17)	0.66	1.78	(0.53, 6.01)	0.35
Preenrollment followup (per year)	1.09	(0.78, 1.52)	0.61	1.08	(0.75, 1.55)	0.68	0.88	(0.42, 1.83)	0.73
Vertical c/d ratio at enrollment (per unit)	2.98	(0.16, 57.01)	0.47	3.98	(0.11, 138.27)	0.44	2.95	(0.03, 311.79)	0.65
Mean HRT topography SD at baseline (per 1 µm)	1.04	(0.98, 1.11)	0.17	1.04	(0.97, 1.11)	0.27	1.05	(0.95, 1.15)	0.35

MRA: moorfields regression analysis; OHT: ocular hypertension; CCT: central corneal thickness; MD: mean deviation; PSD: pattern standard deviation; VFI: visual field index; c/d: cup/disc; HR: hazard ratio; SD: standard deviation; CI: confidence interval; dots (.) denote nonestimable quantities.

**Table 4:** Median times to clinical (ONH stereophotograph and/or Visual Field) progression for TCA-stable and TCA-progressed eyes throughout the whole followup.

Time (months)	Median (IQR)				
	*n*	TCA-stable eyes (*n* = 13)	*n*	TCA-progressed eyes (*n* = 48)	*P* value
Baseline to ONH SPG and/or VF progression	5	7.42 (6.88, 7.73)	11	7.94 (5.12, 8.52)	0.20
Baseline to ONH SPG progression	4	7.15 (6.84, 7.56)	9	7.94 (5.24, 8.74)	0.20
Baseline to VF progression	1	7.73 (7.73, 7.73)	5	5.01 (4.76, 8.07)	0.20

Enrollment to ONH SPG and/or VF progression	5	3.90 (3.36, 4.11)	11	2.31 (1.85, 3.51)	0.04
Enrollment to ONH SPG progression	4	3.63 (3.35, 3.95)	9	2.81 (1.96, 4.01)	0.20
Enrollment to VF progression	1	5.69 (5.69, 5.69)	5	1.85 (1.75, 2.18)	0.005

ONH: optic nerve head; TCA: topography change analysis; SPG: stereophotograph; VF: visual field; IQR: interquartile ranges; dots (.) denote nonestimable quantities.

**Table 5:** Kaplan-Meier cumulative probabilities of stereophotograph (SPG) and/or visual field (VF) progression, SPG only progression, and VF only progression, from recruitment, by topography change analysis (TCA) status.

Stereophotograph and/or Visual Fields			Stereophotographs		Visual Fields	
Time from enrolment (months)	TCA-stable Mean (95% CI)	TCA-progressed Mean (95% CI)	TCA-stable Mean (95% CI)	TCA-progressed Mean (95% CI)	TCA-stable Mean (95% CI)	TCA-progressed Mean (95% CI)
0	0.0 (.,.)	0.0 (.,.)	0.0 (.,.)	0.0 (.,.)	0.0 (.,.)	0.0 (.,.)
12	0.0 (.,.)	0.0 (.,.)	0.0 (.,.)	0.0 (.,.)	0.0 (.,.)	0.0 (.,.)
24	0.0 (.,.)	10.7 (4.1, 26.0)	0.0 (.,.)	7.8 (2.6, 22.3)	0.0 (.,.)	8.0 (2.6, 22.7)
36	0.0 (.,.)	18.0 (9.0, 34.0)	0.0 (.,.)	12.7 (5.5, 27.9)	0.0 (.,.)	10.5 (4.1, 25.5)
48	26.0 (9.0, 61.8)	20.5 (10.8, 36.7)	26.0 (9.0, 61.8)	15.3 (7.2, 30.9)	0.0 (.,.)	10.5 (4.1, 25.5)
60	36.5 (15.2, 71.6)	24.4 (13.3, 42.2)	36.5 (15.2, 71.6)	19.5 (9.6, 37.4)	00 (.,.)	10.5 (4.1, 25.5)

Dots (.) denote nonestimable quantities.
